# Marburg virus in Ghana: A public health threat to Ghanaians and to Africans

**DOI:** 10.1002/puh2.32

**Published:** 2022-11-02

**Authors:** Salomey Asaah Denkyira, Ridwan Olamilekan Adesola, Ibrahim Idris, Kwasi Yelarge, Kwame Tieku Asaaseasa, Cynthia Amaning Danquah, Emmanuel Opuni

**Affiliations:** ^1^ Faculty of Pharmacy and Pharmaceutical Sciences Kwame Nkrumah University of Science and Technology Kumasi Ghana; ^2^ Department of Veterinary Medicine Faculty of Veterinary Medicine University of Ibadan Ibadan Nigeria; ^3^ Department of Veterinary Medicine Faculty of Veterinary Medicine Usmanu Danfodiyo University Sokoto Nigeria; ^4^ Department of Health Policy London School of Economics and Political Science London UK

**Keywords:** Africa, Ghana, haemorrhagic fever, Marburg virus disease, outbreak

## Abstract

The Marburg virus disease (MVD) outbreak in Ghana and the re‐emergence of other infectious diseases in Africa require a unified effort to fight these highly infectious health threats.  Although no new cases have been reported after the three MVD confirmed cases, Ghana Health Service (GHS), and Health Authorities of neighbouring countries are on the alert to identify any new cases and deal with them at the earliest. Public health measures, however, inadequate, are in place in Ghana.   Varied challenges remain in the fight against these outbreaks. All known public health measures must be brought to bear in the fight. African governments must resource the African Centre for Diseases Control and Prevention, in its efforts to augment the various country‐level health services in the fight against the threats. African governments, citizens, development partners, and foreign governments must commit to fighting these diseases before they turn into pandemics.

## INTRODUCTION

Marburg virus disease (MVD) is a severe haemorrhagic fever which affects both human and non‐human primates. The disease‐causing virus, formerly known as the Marburg Haemorrhagic Fever (MHF) virus, belongs to the same family as Ebola‐*Filoviridae* [1]. The disease is characterized by fever, general malaise, bleeding from the nose and mouth, subconjunctival bleeding, diarrhoea, and vomiting [2]. The incubation period in humans ranges from 2 to 21 days, with an average incubation period of 5–9 days [3]. Usually, fatalities typically occur between 8 and 16 days after the onset of symptoms [4]. Marburg virus transmission can occur through mucosal surfaces and breaks or abrasions of the skin, as well as through parenteral introduction. Direct contact with infected humans or animals is the most common source of infection and occurs via direct contact with blood or other secretions/excretions (e.g., saliva, sweat, stool, urine, tears or breast milk) of an infected person [5]. Studies have also demonstrated that MVD is highly infectious and lethal following experimental aerosol exposure [6].

Unfortunately, there is no specific therapy for MVD available, treatment currently involves palliative management of symptoms, including pain management, antibiotics and antipyretics to reduce fever, prevent and treat secondary infections and supportive care measures such as maintenance of blood volume and electrolyte balance [4].

In July 2022, the Ghana Health Service (GHS) reported three confirmed cases of MVD leading to health security threats for Ghana and Africa. About 57 years ago in Germany, MVD was reported in humans. This has been followed by outbreaks in African countries including Angola, Uganda, Zimbabwe, and Kenya (Table 1), other outbreaks include the United States, Russia, and the Netherlands [7]. The case fatality is between 24% and 88% [8]. In this piece, we report on the outbreak, and its impact on Africa and offer some recommendations. As the disease is highly infectious with very high case fatality, it poses a health security threat to Ghanaians, neighbouring African countries, and the world. There are many challenges that Ghana and African countries face in the MVD epidemic. Public Health responses are in place since the confirmation with the GHS coordinating the efforts to contain the disease with the help of partners. Other infectious diseases like Ebola, Monkeypox, leptospirosis, etc are re‐emerging in Africa coupled with COVID‐19, which is impacting so much on African economies. With these kinds of highly infectious diseases breaking out in fragile health systems in Africa, there is a need to collaborate to fight them together as has already been identified by the African Union. International partners and governments should also contribute their quota to fight these infectious diseases to avoid pandemics.

**TABLE 1:** Historical number of outbreaks in different countries in Africa

**African countries**	**Number of outbreaks**	**Year of outbreaks**
Serbia	1	1967 [9]
South Africa	1	1975 [10]
Democratic Republic of Congo	1	1998–2000 [11]
Angola	1	2004–2005 [12]
Guinea	1	2021 [13]
Ghana	1	2022 [8]
Kenya	2	1980, 1987 [14]
Uganda	6	2007, 2008, 2012, 2014, 2017 [15]

### Account of the outbreak

**FIGURE 1:**
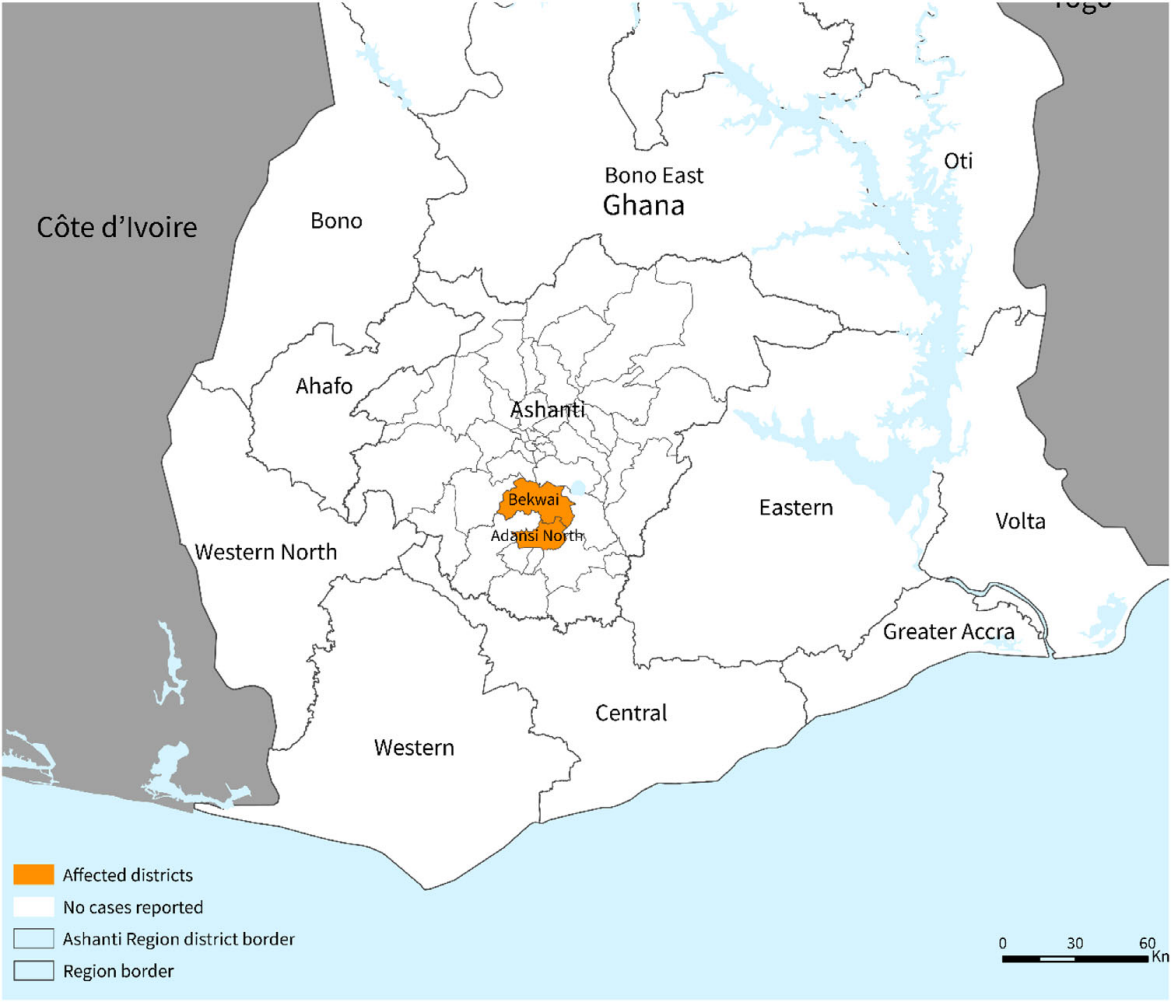
Map of Ghana with a region where the MVD has been confirmed [8]

A suspected case of Viral Haemorrhagic Fever (VHF) was reported to the Health Authorities in the Ashanti region of Ghana in June 2022 [16]. This was a 26‐year‐old male farmer in the Ashanti region. He sought medical care at a Hospital in the Ashanti region (Figure 1) on June 26, 2022, and died the next day. His body was transported to the Savanna Region in the North of Ghana; and was traditionally buried before laboratory confirmation of the infection, by the Institut Pasteur in Dakar, Senegal, was available [16]. This is the index case. Following his infection, his 24‐year‐old wife and 1‐year‐old son were also confirmed to be infected with MVD [7]. Therefore, there are three confirmed cases (involving a nuclear family) of which two have died‐the index case and his 1‐year‐old son.

### Health security issue

The outbreak initially posed a higher level of health security threat in Ghana, but it has been lowered [9, 18]. Similar to Ebola, Marburg spreads from human to human by direct contact (through broken skin or mucous membrane) with blood, secretions, organs or other bodily fluids of infected people and with surfaces and materials (like clothes, beddings) contaminated with these fluids [16]. Traditionally, Ghanaians bathe the corpse before burial; and mourners may touch the corpse while crying during the laying‐in state. People also shake hands and hug each other while mourning etc. These traditional practices can lead to a wider outbreak. The 26‐year‐old man travelled to the Western Region near Ivory Coast. His corpse was transported to the Savanna Region to be buried‐which is close to Burkina Faso and Ivory Coast borders. He was buried before the laboratory results were released [16] and the necessary public health procedures were not followed in handling the corpse, possibly exposing others to the diseases, including neighbouring Ivory Coast and Burkina Faso. Furthermore, the location of the index case could have facilitated its spread to parts of the country, given its central location in Ghana and its openness to large commercial activities in Ghana and its neighbours. The close contacts of the infected numbering 198 were quarantined. Updated data shows that 118 of them have been discharged following 21 days of no symptoms/negative test results. The remaining are waiting for the 21 days incubation period to expire. There is no pending confirmed case in Ghana currently, but surveillance and other public health measures are ongoing to contain any health security threats [17]. Currently, the health security threat is no longer high for Ghana.

### Public health response

After the outbreak of the MVD, Public Health measures have been employed by the GHS, supported by other Partners. The GHS isolated individuals that were in contact with the confirmed cases [17, 18]. Further, the Ashanti Regional Directorate of GHS has established coordination mechanisms and responses for the Marburg cases, with epidemiological investigations and follow‐up, and sensitization activities among others [16]. The WHO had mobilized public health support to Ghana in terms of pieces of equipment and chemical reagents provided to the Nuguchi Memorial Institute for Medical Research (NMIMR) for diagnosis of the virus, in addition to the deployment of technical experts to help contain the disease. Community‐based surveillance volunteers have been oriented to enhance surveillance at the community level. Ivory Coast and Burkina Faso authorities have also been informed to prepare for any outbreak [16]. Other public health measures are in place, coordinated by GHS, to contain the MVD.

### Challenges

Firstly, the source of infection is yet to be identified. Preliminary investigation revealed that the victims had no history of contact with an infected animal or human. Notwithstanding, the current information indicates that the 26‐year‐old was a miner; mining caves can be abodes for fruit bats which are known transmitters of viruses from animals to humans. Therefore, it is possible that the index case contracted the disease from his mining activities. It is possible that human‐to‐human transmission happened between the index case and his immediate family. Again, it is possible that the family of the index case had contact with bushmeat (meat of game) which contained the infection. In another development, the genomic sequence analysis from Institut Pasteur in Dakar, Senegal‐of the Marburg in Ghana suggests that it is related to the case reported in Guinea in 2021. More analysis is needed to understand this link and the public health implications.

Furthermore, the resource‐constrained healthcare system in Ghana poses a major challenge. For example, a lack of testing centres, treatment centres, isolation centres, supplies, etc., and weak public health implementation render the nation vulnerable in combating the MVD epidemic [19]. The situation is not different in other African countries [20]. Another challenge is health financing issues. In Ghana, the government is a major financier of healthcare through the National Health Insurance Scheme (NHIS) and taxes. The NHIS is likely to be affected, as many subscribers are not likely to renew their health insurance policies because they fear contracting the MVD when they visit health facilities [18]. Additionally, the non‐attendance at health facilities by health consumers will impact out‐of‐pocket payments and the hospitals’ ability to generate sufficient internal resources for their operations‐hence a major setback in the fight against MVD.

The government has committed resources to combat the MVD [21]; the government is likely to divert more resources should there be an MVD epidemic, which will not augur well for other health needs of equal importance. Moreso, the cultural handling of the sick and the dead in Ghana can pose a challenge to the containment of the outbreak. Some may trace sicknesses to spiritual causes (causation theory). Following this, some may not adhere to public health guidelines in handling the sick and the dead. There is also a tendency to look for spiritual solutions. This could create bottlenecks for GHS and the partners in their efforts.

### Impact on Africa

The Marburg virus provides an impetus for Africa to continue strengthening its ability to address and combat epidemics. There have already been strides in developing these systems, particularly by the Africa centre for disease prevention and control (CDC), as the continent has been impacted by many re‐emerging epidemics. Given the continuous emergence of different viruses that have the propensity to develop into pandemics within the continent, African governments should work together, collaborate and pull resources together to finance and contain the disease. Efforts such as the pronouncement by the African Union, particularly to increase initiatives and funding for CDC Africa, should be harnessed. African countries should share information, capabilities, and resources and continue to comply with international health regulations [22].

### Recommendations

Following the lead provided by the WHO [16], the GHS should continue its public health measures to ensure any new cases are identified and addressed at the earliest. Information toward reducing transmission, public infection prevention and control measures, and stigmatization avoidance should be disseminated to the public through local radio stations, district information services, traditional rulers, religious bodies, and labour unions among others. All known protective measures should be adhered to.  All suspected cases and confirmed cases should be isolated at the earliest instance. Medical staff must be reoriented in the professional diagnoses and handling of suspected and confirmed cases. Enhanced surveillance, testing of suspected cases promptly, and reporting of accurate data for policy analysis and ongoing decision‐making will be helpful. All confirmed cases should be handled in a hospital facility and all deaths of both suspected and confirmed MVD cases should be buried by public health guidance. Control and prevention of zoonotic diseases require different specialists to tackle the issue appropriately. All known prevention and control measures should be brought to bear in a bid to contain the current MVD outbreak in Ghana.

## CONCLUSION

There has been an outbreak of MVD in Africa again, leading to a fresh call for Africa to organise a united force to fight these recurring health threats.  As a rare, highly infectious, and fatal viral disease without licensed treatment medication, the MVD's threat to health security is not in doubt. Though public health measures have been employed to contain the disease in Ghana, challenges common to other African countries remain. All the public health measures known should be brought to bear in fighting the MVD. African governments should resource the Africa CDC to fight the threat. An international response will be required to fight the virus and other infectious diseases re‐emerging in Africa to avoid the spread to other parts of the world. In addition to this, there is a need for radical solutions to the major challenges that pose a risk to the healthcare system of Ghana and Africa at large.

## AUTHOR CONTRIBUTIONS

Research conceptualization and design: ROA and II; Methodology: Ibrahim Idris, Ridwan Olamilekan Adesola, Salomey Asaah Denkyira, Kwasi Yelarge, Kwame Tieku Asaaseasa, Cynthia Amaning Danquah, and Emmanuel Opuni; Data acquisition: Ibrahim Idris, Ridwan Olamilekan Adesola, Salomey Asaah Denkyira, Kwasi Yelarge, Kwame Tieku Asaaseasa, Cynthia Amaning Danquah, and Emmanuel Opuni; Draft manuscript preparation and revision: Ibrahim Idris, Ridwan Olamilekan Adesola, Salomey Asaah Denkyira, Kwasi Yelarge, Kwame Tieku Asaaseasa, Cynthia Amaning Danquah, and Emmanuel Opuni. All the authors read and approved the final draft before submission.

## CONFLICT OF INTEREST

Dr. Danquah, Cynthia Amaning is an Editorial Board member of Public Health Challenges and also a co‐author of this article. To minimize bias, she was excluded from all editorial decision‐making related to the acceptance of this article for publication.

## ETHICS STATEMENT

Not applicable.

## Data Availability

No primary data gathering was conducted and no databases were used. This is a literature‐based article.
